# Toxicity of a Novel Herbomineral Preparation Las01 on Human Cancer Cell Lines and Its Safety Profile in Humans and Animals

**DOI:** 10.1155/2012/948375

**Published:** 2012-08-30

**Authors:** Saba Sheikh, Ashok Srivastava, Rajesh Tripathi, Shalini Tripathi, V. P. Trivedi, R. C. Saxena

**Affiliations:** R & D Division Lavanya Ayurveda Biotech Park, Lavanya Ayurvedic Hospital and Cancer Research Centre, Chinhat, Dewa Road, Lucknow 226016, India

## Abstract

Polyhedral formulations based on Rasayana therapy described in Charaka Samhita showed remarkable improvement in quality of life of various cancer patients who have been found to be refractory or poor responders to modern chemotherapy and radiation treatment. One of the most recent novel herbomineral preparation, Las01 prepared absolutely as per the instruction given in the ancient Ayurvedic literature has been found to be effective as a potent anticancer drug in the human cell lines, the MCF-7 and Hela cancer cell lines. This novel preparation of Las01 is also found to be devoid of toxicity both in animals as well as in human subjects, which is the main drawback of chemotherapeutic agents used in modern system of medicine. Our results warrant multicentric clinical trials on a large scale which seems to be a future promising drug to cure incurables cancer patients.

## 1. Introduction

Cancer has become one of the biggest challenges to the scientific community over the world, and despite development of drugs and other modalities for treatment of Cancer, however there are complexities at every level of treatment. Cancer chemotherapy is associated with many unwanted side effects such as nausea, loss of taste, lethargy, loss of hair, loss of libido, immunosuppression and myelosuppression, and tumorogenesis [1]. Thus there is need to find out relatively safe, effective, and economical solution for cancer. The scientific community is looking at traditional holistic system of the medicines for treatment of cancer. Herbomineral therapeutics is one of the most promising areas of treating diseases like cancer. The branch of Ayurveda deals with medicinal properties of herbometallic and herbomineral preparations known as Rasa shastra and the drugs which are used are known as Rasa Aushadhi. They were more popular during the period of Lord Buddha due to its faster relief, lesser and convenient doses, and mysterious efficacy as compared to only herbal drugs. Various constituents of Herbomineral compounds such as gold, silver, lead, iron, and arsenic are never used in the raw form as their raw form causes poisonous and toxic effects [2]. 

Modern science has now revealed that this process of anaerobic cooking used in preparing Herbomineral drugs converts the toxic mega particles of metal into safe and efficacious nanoparticles and even smaller picoparticles which explains the usefulness of Herbomineral drugs as effective medicines for cancer as prophylactic, palliative, curative, and supportive medicaments. Therefore, Rasayana therapy and its role in cancer management are being screened in almost all leading Ayurveda research institutes in this country. Polyhedral formulations based on Rasayana therapy described in Charaka Samhita showed remarkable improvement in quality of life of various cancer patients who were treated earlier with chemotherapy and radiotherapy. It was also effective in overcoming the side effects of chemotherapy and radiation such as hair loss, weight loss, stomatitis, and xerostomia [3, 4]. In the present study the safety and toxicity of Las01 were checked in animals as well as human beings and the therapeutic efficacy of its anticancer activity has been checked on breast cancer cell line MCF-7 and HeLa cervical cancer cell line job1041.

## 2. Material and Methods

### 2.1. Las01 Herbomineral Anticancer

The drug used in the present study was Las01 a Herbomineral preparation prepared by Lavanya Ayurvedic Hospital and Cancer Research Centre by its own manufacturing unit exactly as per the instructions laid down in our ancient Ayurvedic literature [5]. Accordingly Las01 preparation contains a number of herbs and different types of inorganic minerals such as mercury which has been extensively purified through 75 steps as per Kupipakva Rasayana technique yielding an anticancer drug in the form of bhasma. This Herbomineral drug was standardized by the use of physiochemical properties and transmission electron microscopy.

### 2.2. Animals, Feed, Dose, and Experiment

Charles-Foster strains of albino rats of either sex with an average body wt 150–200 gm were used in the experiment. 16 animals were taken in each group for acute toxicity study. Rats were randomly divided into four groups and one group served as control (2 animals/group of either sex) for 14 days in order to study the acute toxicity. For chronic toxicity study 36 rats were randomly divided into four groups and group one served as control and was kept normal (3 animals/group of either sex) for 90 days in order to study the chronic toxicity. The Sagar Institute of Technology and Management Lucknow animal house facility was used; all animal experiments were conducted after getting approval from institutional animal ethics committee of the institute. The standard animal conditions of room temperature 21 ± 20°C, relative humidity 60 ± 10%, and 12 h light/dark cycle were maintained. The commercial pellet diet and reverse osmosis water for rats were available *ad libitum*. The dose for experimental study of the test drug Las01 was calculated by extrapolating the human dose (1000 mg/day) to animal dose (88.02 mg/kg normal dose = 1x; 880.2 mg/Kg body wt. = Las01 10x; 3520.80 mg/Kg body wt. =Las01 40x) based on the body surface area ratio and the drug was administered orally to the animals [6].

**Figure 1:**
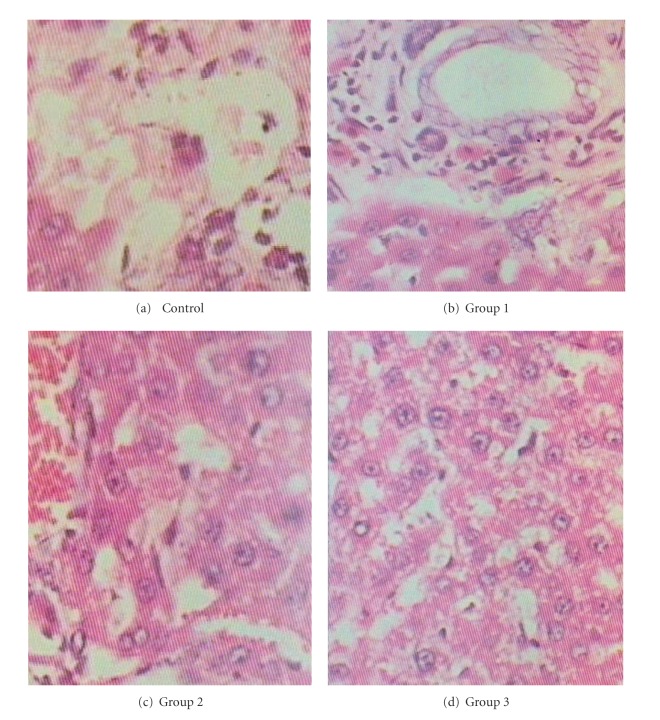
Histopathological pictures of liver after 90-day exposure of Las01. Magnification (40x). (a) Control normal Liver. (b) Group 1 treated liver (88.02 mg/Kg body wt. = Las01 1x). (c) Group 2 treated liver (880.2 mg/Kg body wt. = Las01 10x). (d) Group 3 treated liver (3520.80 mg/Kg body wt. =Las01 40x).

**Figure 2:**
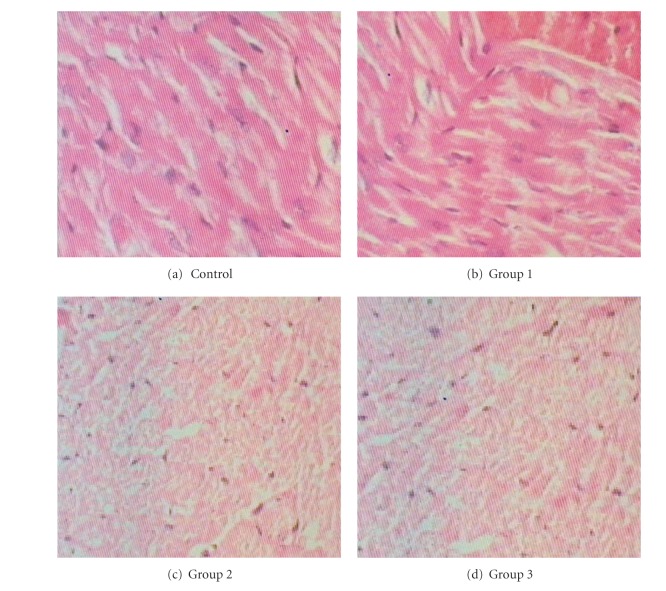
Histopathological pictures of heart after-90 day exposure of Las01. Magnification (40x). (a) Control normal Heart. (b) Group 1 treated heart (88.02 mg/Kg body wt. = Las01 1x). (c) Group 2 treated heart (880.2 mg/Kg body wt. = Las01 10x). (d) Group 3 treated heart (3520.80 mg/Kg body wt. =Las01 40x).

**Figure 3:**
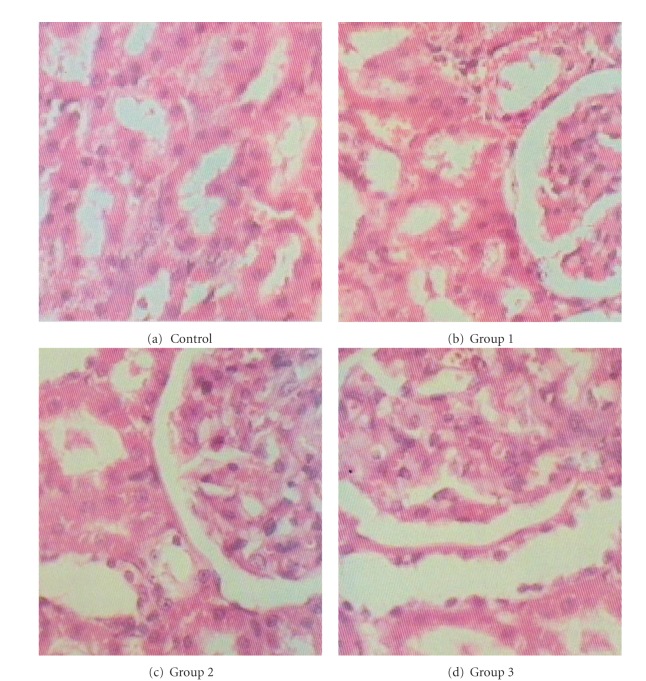
Histopathological pictures of kidney after 90-day exposure of Las01. Magnification (40x). (a) Control normal Kidney. (b) Group 1 treated kidney (88.02 mg/Kg body wt. = Las01 1x). (c) Group 2 treated kidney (880.2 mg/Kg body wt. = Las01 10x). (d) Group 3 treated kidney (3520·80 mg/Kg body wt. =Las01 40x).

The study was divided into two phases, acute and subchronic. In acute toxicity the animals were sacrificed on the 14th day while in subchronic studies they were sacrificed on the 90th day. During autopsy the shape, size, and colours of internal organ were visually observed for any signs of lesions. The blood was collected by cardiac puncture method and collected into EDTA-K3 and clot activator non-VAC. tubes of LabTech Company for haematological and biochemical analysis, respectively. Haematological parameters were checked by automated cell counter and haemoglobin by cyanmethemoglobin method [7] by blood. Serum was separated through centrifugation of blood at 3000 rmp for 10 minutes for biochemical analysis and was performed using prepared kits from Roche Diagnostic reagents. Serum glutamate oxaloacetate transaminase (SGOT), serum glutamate pyruvate transaminase (SGPT), and alkaline phosphatase (ALP) were estimated by (International Federation of Clinical Chemistry) IFCC Method [8], bilirubin total and direct by Jen Drasik and Grof method, creatinine by Jaffe and Kinetic method, blood urea nitrogen by Glyceraldehyde dehydrogenase method [9], total protein by Biuret method, and albumin and globulin by Bromocresol Green method. Serum sodium and potassium were estimated by Biolyte 2000 ion elective electrolyte analyzer [10]. Histopathological analysis of the liver (Figure 1), kidney (Figure 3), heart (Figure 2), and lung was performed by cutting 2 mm section of the tissue fixed in 10% formaldehyde by a microtome and further staining with haematoxylin and eosin and was examined by a pathologist.

### 2.3. Safety Profile of Las01 in 25 Patients of Different Cancers

Lavanya Ayurvedic Hospital has been treating cancer patients with Las01 Herbomineral for the last several years. In the present study blood from 25 patients was taken for hematological and biochemical (hepatic and renal) toxicity of Las01 preparation. After obtaining written informed consent forms from patients and clearance from IEC, blood was analyzed before and after 1-year treatment of cancer patient by Las01 Herbomineral preparation.

### 2.4. In Vitro Anticancerous Activity

#### 2.4.1. Cancer Cell Lines and Culture Conditions

Human HeLa cervical cancer cell lines and MCF-7 breast cancer cell lines (adenocarcinoma) were obtained from National Centre for Cell Sciences (NCCS) Pune, India. Both cell lines were cultured as monolayer cultures in Dulbecco's modified Eagle's medium (DMEM) containing 10% fetal bovine serum and antibiotics (100 units/mL penicillin and 100 mg L^−1^ streptomycin) in a humidified atmosphere of 5% CO_2_ at 37°C in T-75 flasks and were subcultured twice a week.

#### 2.4.2. Cytotoxicity Assay

The cytotoxic effect of Las01 was assessed in human cervical cancer HeLa and MCF-7 breast cancer cell lines (adenocarcinoma) cells by the MTT [3-(4,5-dimethylthiazol-2-yl)-2,5-diphenyl tetrazolium bromide] assay [11]. Briefly, cells were seeded at a number of 2 × 10^4^ cells per well on 96-well plates (200 *μ*L/well) in triplicates and exposed to ethanol dissolved in Las01 different concentrations for 48 h (25 mg/L–500 mg/L). At the end of the treatment, the medium was removed and cells were incubated with 20 *μ*L of MTT (5 mg/mL in PBS) in fresh medium for 4 h at 37°C. After four hours, formazan crystals, formed by mitochondrial reduction of MTT, were solubilized in DMSO (150 *μ*L/well) and the absorbance was read at 570 nm after 10 min incubation on the iMark Microplate Reader (Bio-Rad, USA). Percent of inhibition of cytotoxicity was calculated as a fraction of control (without Las01) and the cytotoxicity of Las01 was expressed as % inhibition.

#### 2.4.3. LDH Assay

Cytotoxic property of Las01 was also assessed by lactate dehydrogenase (LDH) leakage into the culture medium. Following exposure to the Las01, the culture medium was aspirated and centrifuged at 3000 rpm for 5 min in order to obtain a cell-free supernatant. The activity of LDH in the medium supernatant was determined using a commercially available *CytoScan *by using* LDH* Cytotoxicity Assay Kit. Percent of inhibition of cytotoxicity was calculated as a fraction of control (without Las01) and the cytotoxicity of Las01 was expressed as IC50.

#### 2.4.4. Viability Staining by Trypan Blue Dye Exclusion Method

Cytotoxic activities of Las01 were analyzed by trypan blue dye exclusion method adopted by Ian Freshney, 1994. Cell lines in exponential growth phases were washed with PBS solution and trypsinized and resuspended in complete culture media. Cells were plated at 5000 cells/well in 6-well plates and incubated for 24 hrs during which partial monolayer was formed. After incubation the cells were exposed to various concentrations of the drug. The control well received only maintained media. The plates were incubated at 37°C in 5% CO_2_ incubator for a period of 24 hrs. Morphological changes were examined using inverted microscope and compared with the cells serving as control. At the end of 24 hrs incubation cell viability was determined.

#### 2.4.5. Determination of Apoptosis and Necrosis

Apoptotic and necrotic cells were analyzed with double staining to quantify the number of apoptotic cells in culture on the basis of scoring of apoptotic cell nuclei. HeLa cells (5000 cells per well) were placed in DMEM by using 6-well plates and treated with different concentrations of functional oligomers (about 0 to 200 *μ*g·mL^−1^ in phenol red free medium) for 24 h period. Both attached and detached cells were collected, then stained with Hoechst dye 33342 (2 *μ*g·mL^−1^), propodium iodide (PI) (1 *μ*g·mL^−1^), and DNAse free-RNAse (100 *μ*g·mL^−1^) for 15 min at room temperature [11]. Necrotic cells were stained red by PI. Necrotic cells lacking plasma membrane integrity and PI dye cross cell membrane, but PI dye does not cross nonnecrotic cell membrane. The number of apoptotic and necrotic cells was determined with DAPI and FITC filters of Fluorescence Inverted Microscope (Leica, Germany).

## 3. Results 

### 3.1. Acute Toxicity

#### 3.1.1. Effect of Las01 on Haematological and Biochemical Parameter

There was no significant change in total WBC count, haemoglobin content, and biochemical parameters in animals treated with Las01 upto 40-fold doses for a period of 14 days (Table 1). All the parameters are within the normal limits and animals did not show any sign of hyperactivity. ALD was not found up to 5 mg of Las01. 

### 3.2. Chronic Toxicity

There was no significant change of WBC count, haemoglobin content, and differential counts. There was no significant alteration in hepatic and renal function parameters. Subchronic administration of these drugs did not produce any alteration in sodium, potassium, chloride, and bicarbonate levels (Table 2). No remarkable histopathological changes were noted in the internal organs of rats receiving these drugs in higher doses. 

### 3.3. *In Vitro* Activity

**Figure 4:**
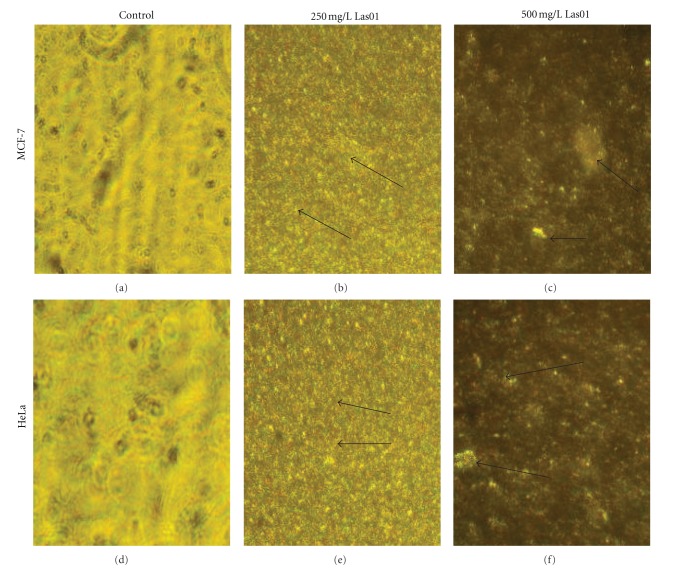


Data on the cytotoxic effects of Las01 using MCF-7 and HeLa cell lines *in vitro *are shown in Table 3. Cytotoxic effects of Las01 on MCF-7 and HeLa cell lines *in vitro *by MTT and LDH methods, respectively, inhibited proliferation of both MCF-7 and HeLa cervical cancer cells in a dose-dependent manner. The cytotoxic effect of Las01 was determined using concentrations ranging from 10 mg L^−1^ to 500 mg L^−1^ for 48 h. After 48 h exposure, Las01 induced concentration-dependent cytotoxic effect about 78% at 500 mg/L in cervical cell lines and 77% at 500 mg/L in MCF-7 breast cancer cell line. Trypan blue was used to stain cells for observing the ratio of viable and dead cells. It was observed that increasing the concentration (10 to 500 mg/L) of Las01 decreases viability of cancer cells. Low concentrations of the Las01 produced low toxic effect on cells whereas at higher concentrations (500 mg/L) there was higher effect in toxicity so that in only 20% and 18% in MCF-7 and HeLa, respectively, viability was observed (see Table 4). The results obtained with double staining of control and treated cells are presented in Figure 4. The control cells fluoresced uniformly green and had normal features. Most of the cells treated with Las01 fluoresced red and indicated apoptotic features such as cell shrinkage, chromatin condensation, nuclear fragmentation, and apoptotic body formation. A few cells indicated necrotic features such as cell swelling and lysis in higher concentration of Las01 1000 mg/L. 

The results obtained with double staining of control and treated cells are presented in Figure 4. The control cells fluoresced uniformly green and had normal features. Most of the cells treated with Las01 fluoresced green and indicated apoptotic features such as cell shrinkage, chromatin condensation, nuclear fragmentation, and apoptotic body formation comparatively less in 250 mg/l than in 500 mg/L.

## 4. Discussion

Ayurveda is a traditional medical system used by the majority of India's 1.1 billion populations. The word Ayurveda literally means science of life. It is time tested and trusted through thousands of years of usage. Ayurveda, from the days of Charaka and Sushruta, was primarily used as medicinal plants for the preparation of therapeutic agents. It is only in the 8th century AD that Indian alchemist Nagrjuna prescribed the use of metals (e.g., mercury, lead, cadmium, iron, and zinc) and minerals (e.g., mica) as medicinal agents. Since these were very effective, quick in action, even in smaller dosage, palatable, and having longer shelf life, the use of minerals and metals became the backbone of Ayurvedic therapeutics [12].

Cancer is a major cause of death or morbidity in human populations [13]. The human body is made up of different cells. Cells divide and multiply as per the need of body system. However, when balance between protooncogenes and tumor suppressor gene is disturbed due to several factors these cells continue to divide needlessly resulting in tumor formation [14]. There is evidence that potentially toxic macro- or microparticles of heavy metals could be nontoxic if these particles are converted into nanoparticles [15]. The search for novel antitumor compounds in phytotherapy is a promising strategy for the prevention of cancers [16]. Cellular proliferation depends on the rates of cell division and death and, thus, many anticancer drugs have been used to prevent cancer cell division in order to inhibit cancer cell proliferation. *In vitro* cytotoxicity assays can be used to predict human toxicity and for the general screening of chemicals [17, 18]. It has been previously reported that different cytotoxicity assays can give different results depending on the test agent used and the cytotoxicity assay employed [19]. Our present study demonstrates not only the efficacy of Las01 in *in vitro* tests on human cervical cancer cell line (HeLa) and breast cancer cell line (adenocarcinoma MCF-7) but also the safety of Las01 on variety of haematological and biochemical parameters both in animal experiments as well as in human beings. Las01 dosage formulation prepared by Lavanya Ayurvedic Hospital and Cancer Research Centre as per the instructions described in our ancient Ayurvedic literature is also supported by the observations of Dasgupta and Cuenca [14, 15]. Our *in vitro* experiments on apoptosis also demonstrate that the characteristic immortal cancer cells are converted into mortal cells indicating carcinostatic as well as carcinocid activity of Las01. In view of the efficacy and safety of Las01, a Herbomineral standardized preparation, its extensive clinical trial is warranted in a variety of carcinomatous conditions. 

## 5. Conclusion

One of the most recent novel Herbomineral preparations, Las01, prepared absolutely as per the instruction given in the ancient Ayurvedic literature has been found to be an effective drug on the human cell lines MCF-7 and HeLa cancer cell line. This novel preparation of Las01 is also found to be devoid of toxicity both in animals as well as in human subjects, which is the main drawback of chemotherapeutic agents used in modern medicine. In view of our encouraging results, multicentric clinical trials are warranted on a large scale which seems to be a novel and future promising drug to cure incurable cancer patients.

## Figures and Tables

**Table 1:** Effect of acute administration of Las01 in rats.

Parameters	Control	Las01		
		Group 1	Group 2	Group 3
Total WBCcount × 10^3^ mm^3^	6293 ± 488	6342 ± 422	6321 ± 230	6234 ± 230
Haemoglobin (gm%)	11.91 ± 1.85	11.82 ± 1.33	11.75 ± 1.0	11.82 ± 1.0
Bilirubin total (mg/dL)	0.31 ± 0.13	0.30 ± 0.12	0.31 ± 0.15	0.32 ± 0.15
Bilirubin direct (mg/dL)	0.24 ± 0.32	0.29 ± 0.27	0.25 ± 0.25	0.27 ± 0.15
SGPT (U/L)	44.8 ± 11.5	45.7 ± 11.1	44.53 ± 12.38	46.11 ± 12.38
SGOT (U/L)	135.4 ± 19.6	139.3 ± 15.26	136.2 ± 17.25	133.4 ± 17.25
ALP (U/L)	161.8 ± 92.1	168.6 ± 93.2	170.7 ± 94.5	163.6 ± 91.5
Albumin (mg/dL)	3.53 ± 0.41	3.35 ± 0.46	3.71 ± 0.32	3.47 ± 0.32
Globulin (mg/dL)	4.03 ± 0.19	4.03 ± 0.36	4.00 ± 0.39	4.12 ± 0.39
Total protein (gm%)	7.56 ± 0.28	7.38 ± 0.22	7.71 ± 0.42	7.59 ± 0.42
BUN (mg/dL)	66.58 ± 7.45	63.13 ± 6.6	66.26 ± 7.48	65.32 ± 7.48
Creatinine (mg/dL)	0.45 ± 0.05	0.46 ± 0.04	0.44 ± 0.04	0.44 ± 0.04
Sodium (mEq/L)	146 ± 4.41	147.5 ± 2.82	144.3 ± 5.6	148.3 ± 5.6
Potassium (mEq/L)	4.15 ± 0.18	4.12 ± 0.39	4.14 ± 0. 23	4.16 ± 0. 23
Chloride (mEq/L)	104 ± 1.18	103 ± 1.68	101.8 ± 3.70	102.9 ± 3.70

**Table 2:** Effect of chronic administration of Las01 in rats.

Parameters	Control	Las01		
		Group 1	Group 2	Group 3
Total WBCcount × 10^3^ mm^3^	7634 ± 520	7293 ± 488	7335 ± 230	7653 ± 422
Haemoglobin (gm%)	13.13 ± 1.0	12.10 ± 2.85	12.65 ± 1.0	13.16 ± 1.23
Bilirubin total (mg/dL)	0.29 ± 0.12	0.28 ± 0.14	0.30 ± 0.02	0.31 ± 0.12
Bilirubin direct (mg/dL)	0.25 ± 0.15	0.21 ± 0.12	0.28 ± 0.15	0.33 ± 0.14
SGPT (U/L)	45.11 ± 11.12	46.8 ± 10.5	45.66 ± 11.35	44.6 ± 12.1
SGOT (U/L)	74.2 ± 12.25	73.1 ± 12.6	74.2 ± 19.33	75.4 ± 14.16
ALP (U/L)	210.2 ± 22.5	218.3 ± 44.1	219.8 ± 42.5	228.2 ± 39.5
Albumin (mg/dL)	3.27 ± 0.32	3.31 ± 0.41	3.26 ± 0.12	3.25 ± 0.41
Globulin (mg/dL)	4.15 ± 0.19	4.11 ± 0.10	4.10 ± 0.28	4.64 ± 0.11
Total protein (gm%)	7.32 ± 0.42	7.42 ± 0.28	7.36 ± 0.42	7.89 ± 0.22
BUN (mg/dL)	65.12 ± 6.29	67.22 ± 6.87	64.26 ± 7.09	65.15 ± 5.8
Creatinine (mg/dL)	0.41 ± 0.02	0.42 ± 0.03	0.46 ± 0.01	0.41 ± 0.06
Sodium (mEq/L)	145.1 ± 2.1	144.4 ± 2.39	143.6 ± 3.5	145.8 ± 2.45
Potassium (mEq/L)	4.8 ± 0. 33	4.9 ± 0.34	4.2 ± 0. 76	4.9 ± 0.01
Chloride (mEq/L)	104.1 ± 1.10	108 ± 1.08	103.5 ± 4.20	104.6 ± 1.34

**Table 3:** Determination of cytotoxicity by MTT assay.

		MCF-7			HeLa	
Laso1 conc. mg/L	Absorbance	% inhibition	% viability	Absorbance	% Inhibition	% viability
10	2.009	4%	95%	2.008	5%	92%
25	2.001	10%	90%	2.002	12%	89%
50	1.991	15%	84%	1.990	16%	80%
100	1.850	35%	64%	1.850	38%	65%
150	1.720	41%	59%	1.719	45%	55%
200	1.562	46%	45%	1.561	47%	42%
250	1.320	51%	48%	1.321	57%	38%
300	1.250	66%	39%	1.252	68%	32%
350	1.110	70%	32%	1.109	70%	29%
400	1.001	72%	30%	1.002	74%	25%
450	1.000	75%	24%	1.001	76%	20%
500	.950	77%.	20%	.951	78%	18%

**Table 4:** Effect of administration of Las01 on biochemical parameters of cancer patients.

	Normal range	Pretreatment	1 year after treatment
LFT			
Serum bilirubin (T)	0.2–1.0 mg/dL	0.75	0.78
Serum bilirubin (D)	0.0–0.3 mg/dL	0.32	0.34
Bilirubin (ID)	0.1–1.0 mg/dL	0.43	0.44
SGOT	0–37 (M) 0–31 (F) lu/L	31	28
SGPT	0–37 (M) 0–31 (F) lu/L	29	30
S-Alkaline phosphatase	100–275 lu/L	192	185

KFT			
Blood urea	15–50 mg%	22.6	22.7
S. uric acid	2.0–6.0 mg%	3.9	3.82
Creatinine	0.0–2.0 mg%	0.6	0.7
RBS	70–150 mg%	75	80
Total protein	6.0–9.0 mg%	7.2	7.1
Albumin	3.0–6.0 gm%	3.2	3.0
Globulin	2.0–5.0 gm%	4.0	4.1

Electrolyte			
Serum sodium	135–145 meq/L	140.2	141.1
Serum potassium	3.0–6.0 meq/L	4.21	5.0
